# Contact Tracing for Imported Case of Middle East Respiratory Syndrome, China, 2015

**DOI:** 10.3201/eid2209.152116

**Published:** 2016-09

**Authors:** Min Kang, Tie Song, Haojie Zhong, Jie Hou, Jun Wang, Jiansen Li, Jie Wu, Jianfeng He, Jinyan Lin, Yonghhui Zhang

**Affiliations:** Guangdong Provincial Center for Disease Control and Prevention, Guangzhou, China

**Keywords:** Middle East respiratory syndrome, coronavirus, viruses, contract tracing, China, respiratory infections, MERS, MERS-CoV

## Abstract

Confirmation of an imported case of infection with Middle East respiratory syndrome coronavirus in China triggered intensive contact tracing and mandatory monitoring. Using a hotline and surveillance video footage was effective for tracing all 110 identified contacts. Contact monitoring detected no secondary transmission of infection in China.

In 2015, South Korea reported the largest outbreak of Middle East respiratory syndrome (MERS) coronavirus (MERS-CoV) infection that has occurred outside the Middle East (*1*). This outbreak caused 186 laboratory-confirmed cases and 36 deaths. Subsequent transmission of MERS-CoV in South Korea was associated with local hospitals and caused 3 second-generation infections (*2*).

Of the 186 MERS cases originating in South Korea, 1 was confirmed by China (*3*). A preliminary report of this patient’s exposure history and onset of illness has been published (*4*). The patient was symptomatic in South Korea and traveled by airplane to Hong Kong, China, and then by 2 consecutive buses to Guangdong, China, on May 26, 2015. In Guangdong, his community visits included 2 hotels, 2 restaurants, and 1 enclosed meeting room. The China health authority isolated the patient on May 28 and confirmed the patient’s illness as an imported case of MERS-CoV infection on May 29. To prevent local spread, we conducted a comprehensive investigation to trace all contacts of this case-patient in mainland China and also conducted mandatory monitoring.

## The Study

Because this research was a part of a public health response, our study did not require formal ethical approval from a medical ethics committee. Contact tracing was initiated immediately after we detected the case-patient. We communicated with the airline and bus operators to collect passenger information and undertook personal interviews in related communities. A hotline was set up, and the case-patient’s travel information was published in the media. We investigated hotline callers and identified suspected contacts. We also reviewed video footage from closed circuit television in hotels and restaurants visited by the case-patient, enabling us to identify contacts and measure duration and distance of exposures. Information about bus passengers was limited; consequently, with help from police departments, we analyzed video footage recorded by public surveillance cameras at bus stations and surrounding communities and traced the whereabouts of related passengers.

We identified 110 contacts in mainland China: 87 (79%) were from mainland China, 11 (10%) from South Korea, 2 (2%) from Hong Kong, 6 (5%) from Taiwan, 3 (3%) from Canada, and 1 (1%) from Japan. Of the 110 contacts, 27 were air travel contacts (passengers onboard the same flight with the case-patient); 24 were land travel contacts (stewards and passengers taking the same buses with the case-patient); and 59 were community contacts (persons who had face-to-face contact with the case-patient or who had direct contact with his belongings in hotels, restaurants, and a meeting room) (Table 1). We found 34 (58%) of the community contacts through personal interviews. The hotline resulted in 16 (59%) air travel contacts and 12 (50%) land travel contacts. Reviewing video helped trace 9 (38%) land travel contacts and 25 (42%) community contacts (Table 2). We located all community, air travel, and land travel contacts within 3 days, 6 days, and 8 days, respectively (Figure).

**Table 1:** Results of contact investigation of a patient with an imported case of Middle East respiratory syndrome coronavirus infection, China, 2015

Category	Contacts, no. (%), N = 110	Close contacts,* no. (%), n = 44	Common contacts, no. (%), n = 66
Sex			
M	59 (54)	34 (77)	25 (38)
F	51 (46)	10 (23)	41 (62)
Age group, y			
17–29	39 (35)	10 (23)	29 (44)
30–59	66 (60)	30 (68)	36 (55)
60–70	5 (5)	4 (9)	1 (2)
Tracing approach			
Personal interview	48 (44)	15 (34)	33 (50)
Hotline	28 (25)	18 (41)	10 (15)
Video reviewing	34 (31)	11 (25)	23 (35)
Contacts			
Air travel	27 (25)	6 (14)	21 (32)
Land travel	24 (22)	24 (54)	0
Community	59 (54)	14 (32)	45 (68)
Management†			
Quarantine in designated facility	89 (84)	40 (100)	49 (74)
Self-monitoring at home	17 (16)	0	17 (26)
Symptoms†			
Symptomatic	3 (3)	2 (5)	1 (2)
Asymptomatic	103 (97)	38 (95)	65 (98)

*Close contacts include 2 from Taiwan and 2 from South Korea that returned to their countries before they were identified. We notified local health authorities about these 4 contacts. †Numbers and percentages for contacts and close contacts exclude 4 contacts who left mainland China before they were identified.

**Table 2:** Results of different contact tracing approaches for patient with an imported case of Middle East respiratory syndrome coronavirus infection, China, 2015

Tracing approaches	Air travel contacts, no. (%), n = 27	Land travel contacts, no. (%), n = 24	Community contacts, no. (%), n = 59
Personal interview	11 (41)	3 (13)	34 (58)
Hotline	16 (59)	12 (50)	0
Video reviewing	0	9 (38)	25 (42)

**Figure:**
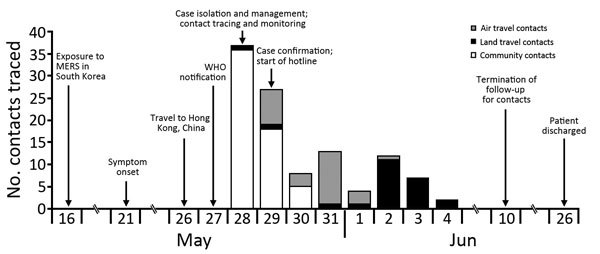
Timeline for imported case of Middle East respiratory syndrome (MERS) coronavirus infection and contact tracing investigation, China, 2015. The case-patient was identified on May 27, 2015, and quarantined beginning in the early morning of May 28, the day contact tracing began. Laboratory testing, which began on May 28, confirmed MERS on May 29, the date of the start of the hotline. WHO, World Health Organization.

Among 44 contacts whom we classified as close contacts, 6 were air travel contacts who had been seated <3 rows from the case-patient on the flight; 24 were land travel contacts; and 14 were community contacts who had prolonged (>15 minutes) face-to-face (<2 m) contact with the case-patient or direct contact with his belongings. We classified the remaining 66 contacts as common contacts. Of the 44 close contacts, 40 were staying in mainland China and were quarantined in designated facilities for 14 days after their last exposure to the case-patient. Public health officials checked body temperatures twice daily and monitored symptoms. The remaining 4 close contacts, 2 from Taiwan and 2 from South Korea, had returned to their countries before they were traced. We notified local health authorities about these 4 contacts. Of 66 common contacts, 49 were quarantined in designated facilities, and 17 conducted self-monitoring at home (an alternative for common contacts) for 14 days after their last exposure to the case-patient. Public health officials visited them daily. During follow-up, fever developed in 1 contact and 2 others had sore throat.

Throat swab samples from 106 contacts and serum samples from 53 were obtained on the first and last days of follow-up. An additional set of specimens was collected from the 3 symptomatic contacts immediately after onset of symptoms. All specimens were tested by real-time reverse transcription PCR, as previously described (*4*). No specimens tested positive for MERS-CoV, and follow-up for contacts ended on June 10, 2015.

From the date of his isolation (May 28) until the date of his discharge (June 26), the case-patient received direct medical care and examination from 73 healthcare workers (HCWs). These HCWs were not considered contacts in our investigation because they all used personal protective equipment, as recommended by the World Health Organization (*5*). The hospital conducted follow-up with all 73 HCWs until 14 days after their last interaction with the case-patient. No HCW was symptomatic during follow-up. Throat swab and serum samples were obtained from all HCWs on day 10 after the case-patient’s admission and on day 14 after his discharge. All specimens tested negative for MERS-CoV by real-time reverse transcription PCR. Follow-up for HCWs ended on July 10, 2015.

## Conclusions

We traced 110 contacts of a patient with an imported case of MERS-CoV infection in China. Follow-up and laboratory testing indicated that no virus transmission occurred among contacts. Because of the timely notification from South Korea and the World Health Organization regarding the MERS-CoV outbreak (*4*), our hospital was able to prepare in advance for admission of the case-patient. No HCWs were infected. Our findings indicate that human-to-human transmission of MERS-CoV is still limited (*6**–**8*).

To minimize risk of local spreading, China health authorities decided to identify and trace all contacts of the initial case-patient and enforce mandatory monitoring. Evidence supports this aggressive policy. First, the MERS outbreak in South Korea, from where the case-patient traveled, was ongoing, and superspreading was observed there (*9**,**10*). Second, the patient was symptomatic and potentially infectious during his travel and stay in China. Previous clusters have been detected in hospitals and households (*11**–**13*), and MERS-CoV transmission might occur in enclosed settings. Moreover, because knowledge about MERS is still limited, cases could have been easily missed initially.

Our approach illustrates the feasibility of multiple complementary practices for contact tracing. Publicizing information and hotlines can facilitate contact tracing and risk communication and helped us identify >50% of the case-patient’s travel contacts. However, we also had to rule out large numbers of false hotline calls that resulted from inaccurate recall and excessive worry. Review of video footage is another active solution for identifying contacts, especially for anonymous contacts. In our investigation, we directly located some bus passengers who resided near the station by reviewing footage from surveillance cameras. Other bus passengers left the station by private cars, which were captured by cameras. Our inquiries into car registration information traced these contacts successfully. Reviewing video footage can also measure a contact’s exposure objectively and quantitatively. Investigators should combine and compare video footage meticulously to gather pieces of information. 

Our contact tracing and monitoring involved challenges. Contacts came from different countries and regions, and sites of their exposures varied. Also, no identity information for bus passengers was available, and privacy issues were concerns. We spent 8 days tracing these passengers, and some had already left China. Furthermore, lack of knowledge about MERS made some contacts less willing to comply with mandatory monitoring. Nevertheless, we traced and monitored all contacts eventually. We suggest combining multiple approaches and data sources beyond ordinary investigation to trace contacts of persons with imported cases of MERS. 
